# Active range of motion of the shoulder: a cross-sectional study of 6635 subjects

**DOI:** 10.1016/j.jseint.2022.09.008

**Published:** 2022-09-30

**Authors:** Glenn S. Fleisig, Jonathan S. Slowik, Matthew Daggett, Marcus A. Rothermich, E. Lyle Cain, Kevin E. Wilk

**Affiliations:** a American Sports Medicine Institute, Birmingham, AL, USA; b Sano Orthopedics, Lee’s Summit, MO, USA; c Andrews Sports Medicine & Orthopaedic Center, Birmingham, AL, USA; d Champion Sports Medicine, Birmingham, AL, USA

**Keywords:** Shoulder, Range of motion, External rotation, Internal rotation, Flexion, Extension, Abduction, Horizontal abduction

## Abstract

**Background:**

Normative data for passive range of motion are well established, but daily living is comprised of active motion. The purpose of this study was to establish normative values for active range of motion of the shoulder across age, sex, and arm. Our hypotheses were that active range of motion of the shoulder (1) decreases with age group, (2) differs between males and females, and (3) differs between the right arm and left arm.

**Methods:**

Shoulder active range of motion was captured with an eight-camera markerless motion capture system. Data were collected for a heterogenous sample of 6635 males and females of all ages. For each subject, 6 shoulder motions were collected with maximum values measured: external rotation, internal rotation, flexion, extension, abduction, and horizontal abduction. Three-way repeated measures analyses were performed, with 2 between-subject factors (age group and sex) and 1 within-subject factor (arm). The unadjusted threshold for statistical significance was α = 0.05.

**Results:**

External rotation decreased with age (approximately 10° decrease from below 30 years to above 60 years). External rotation was approximately 5° greater in the right arm, whereas internal rotation was approximately 5° greater in the left arm. Flexion decreased with age (approximately 15° decrease from below 20 years to above 60 years). For age groups from 10 to 59 years, extension and horizontal abduction were approximately 5° to 10° greater in females than males. Abduction was greater for females than males. Abduction was also greater in younger people (aged 10-29 years) than older people.

**Conclusion:**

In general, active range of motion of the shoulder decreases with age. Sex (male/female) and arm side (right/left) also influence shoulder range of motion.

The anatomy of the shoulder complex allows for a wide range of activities in daily living and athletic activity. Deficiencies in shoulder range of motion can predispose athletes and others to shoulder injury.^12^^,^^22^^,^^23^ Conversely, return to full range of motion can be challenging after surgical or nonsurgical treatment of shoulder injury.^20^^,^^21^^,^^24^ Thus, comparing a person’s shoulder range of motion to normative values of healthy individuals is vital in the prevention and rehabilitation of shoulder injuries.

Numerous studies have reported passive range of motion of the shoulder as defined by the American Academy of Orthopaedic Surgeons,^1^ showing changes across age groups,^2^^,^^4^^,^^8^^,^^11^^,^^13^ sex,^2^^,^^8^^,^^11^ and side-to-side^2^^,^^6^^,^^14^^,^^17^, ^18^, ^19^^,^^21^, ^22^, ^23^ in athletes, patients, and the general population. Although passive range of motion is well established, daily living is comprised of active motion. Unfortunately, diagnosis of shoulder active range of motion has been limited due to the complexity of shoulder motion, the contribution of scapulothoracic motion to shoulder movement, and the use of manual instruments for measurement.^2^^,^^11^ The advent of new technologies may allow us to establish normative values of shoulder active range of motion and improve our ability to screen and prevent shoulder injuries. One such technology approved by the Food and Drug Administration is the DARI markerless motion capture system, which has been used previously to assess lower extremity and full body movements in relation to injury risk and rehabilitation.^3^^,^^5^^,^^7^^,^^9^ This system uses 8 cameras and software-based algorithms to measure active range of motion of each joint.

The purpose of this study was to use markerless motion capture to establish normative values for active range of motion of the shoulder across age, sex, and arm. Our hypotheses were that (1) normative values of shoulder range of motion would decrease with age, (2) there would be differences between males and females, and (3) there would be differences in range of motion between the right and left arm as most individuals are right handed.

## Methods

Sterling Institutional Review Board determined this retrospective analysis of anonymous, previously collected clinical data to be exempt from institutional review board review. From 2018 to 2022, an active range of motion data were collected for patients, athletes, and clients at 51 facilities throughout the United States via multicamera markerless motion capture technology (DARI Motion, Overland Park, KS, USA). At each facility, a motion capture system collected data with 8 cameras electronically synchronized to their mainframe computer. The markerless system used Captury Live (The Captury GmbH, Saarbrücken, Germany) tracking software, which implements methods previously described.^16^ Both the human body and image domain were represented by Sums of spatial Gaussians. The skeletal motion was estimated by optimizing a continuous and differentiable model-to-image similarity measure. While in the work of Stoll et al,^16^ the similarity measure was based on color similarity; the current approach combines the color similarity with a term computed by a Deep Convolutional Neural Network. The DARI markerless system has been used in several recent studies to measure active range of motion for a variety of activities.^3^^,^^5^^,^^7^^,^^9^^,^^10^^,^^15^ One of these studies compared the markerless motion data to a “gold standard” marker-based data and showed similar consistency of joint angle calculations for each system, although the magnitudes of angle measurements differed between the 2 systems.^10^

For each subject, 6 shoulder motions were collected with maximum values measured: external rotation, internal rotation, flexion, extension, abduction, and horizontal abduction. Instructions for the 6 motions are shown in Table I.

**Table I:** Instructions explained and demonstrated to subjects.

Shoulder abduction
Stand tall with arms at sides, elbows straight, and palms facing forward,
Raise arms laterally until overhead,
Return arms to starting position,
Maintain straight elbows and palms forward throughout movement.
Shoulder horizontal abduction
Stand tall with arms extended forward at shoulder height and palms facing each other,
Pull arms away from each other through the shoulders,
Go as far back as possible,
Return to the starting position.
Shoulder internal/external rotation
Stand tall with the shoulders and elbows at 90 degrees, and palms facing down,
Rotate arms up and back as far as possible,
Then rotate arms down and back as far as possible,
Return to the starting position.
Shoulder flexion/extension
Stand tall with arms at sides and palms facing in toward the body,
Raise arms up and back as far as possible,
Then bring arms down and back as far as possible,
Return to the starting position.

To eliminate a weighting bias from multiple entries from some subjects, only one capture for each participant was included in the analyses. Data from all facilities were deidentified before the researchers receiving them for use in the present study.

Subjects were categorized by their sex (male or female) and by their age group (10-19, 20-29, 30-39, 40-49, 50-59, 60-69, or >69 years of age). For each shoulder parameter, a 3-way repeated measures analysis was performed, with 2 between-subjects factors (age group and sex) and 1 within-subject factor (arm). If there was a significant interaction effect, associated main effects were not reported, with subsequent analyses looking at simple main effects. All pairwise comparisons included a Bonferroni adjustment for multiple comparisons. For all analyses, the unadjusted threshold for statistical significance was set at α = 0.05. Partial eta-squared (η_p_^2^) was reported as a measure of the effect size, with the standard minimum thresholds of 0.01, 0.06, and 0.14 used to determine small, medium, and large effect sizes, respectively.

## Results

**Figure 1:**
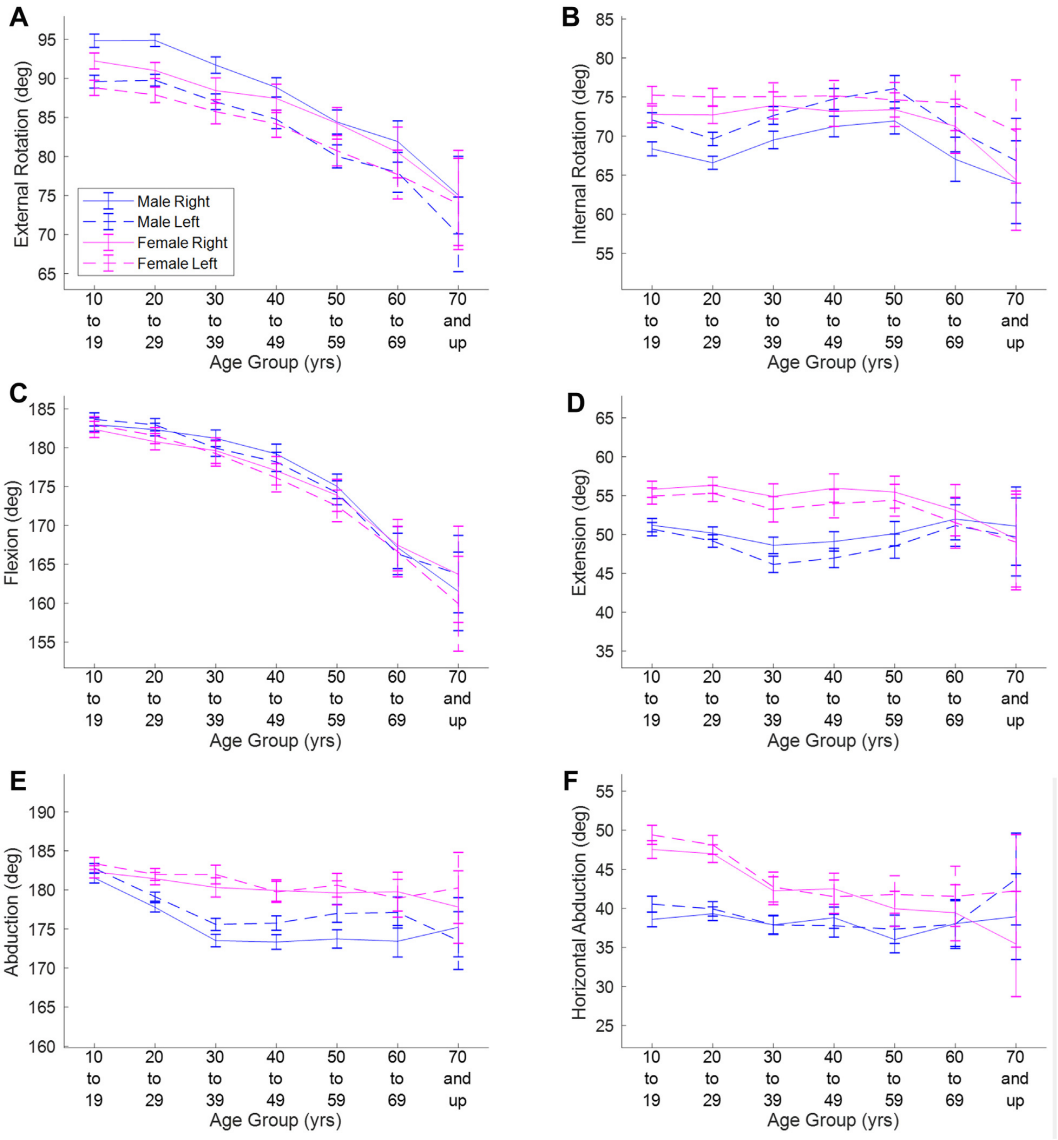
Maximum shoulder angle vs. age. Blue lines correspond to males, whereas pink lines correspond to females. Solid lines correspond to right arms, whereas dashed lines correspond to left arms. Error bars denote 95% confidence intervals.

Data were captured for 6635 individuals at athletic team and performance centers (n = 3980 individuals), health care centers (n = 2142), wellness facilities (n = 327), and military bases (n = 186). The resulting number of subjects in each age-sex combination is provided in Table II. The mean values and confidence intervals for each shoulder motion are presented in Fig. 1 and Table A-1 (in Appendix A). Data are shown for each sex (male and female) and each arm (right and left) for each age group. Significant differences between age, sex, and arm groups are shown in the tables in Appendix B.

**Table II:** Number of participants in each age group for males and females.

	10-19 yr	20-29 yr	30-39 yr	40-49 yr	50-59 yr	60-69 yr	≥70 yr	Total
Male	1129	1321	748	538	342	116	33	4227
Female	778	772	311	250	198	77	22	2408
Total	1907	2093	1059	788	540	193	55	6635

### External rotation

External rotation decreased significantly with age (*P* < .001), and the effect size was small, approaching medium (η_p_^2^ = 0.057). Among the 21 age group pairwise comparisons, 17 showed significant differences (Table B1-1). There was also a statistically significant but trivial interaction effect between sex and arm (*P* < .001, η_p_^2^ = 0.003). Pairwise comparisons revealed that males had greater external rotation than females for right arms, but there was no difference for left arms (Table B1-2). Both males and females had significantly greater external rotation in their right arm (Table B1-3), with a greater difference in males than in females (4.6° vs. 2.8°).

### Internal rotation

There was statistically greater (*P* < .001) internal rotation in the left shoulder than right shoulder (Table B2-1), and the effect size was small (η_p_^2^ = 0.027). There was also a statistically significant interaction but trivial effect between age and sex (*P* = .001, η_p_^2^ = 0.003). Among males, there were statistically significant pairwise comparisons between age groups, but with inconsistency regarding whether the older or younger group in these comparisons had higher value (Table B2-2). In contrast, among females, no age group effect was found (Table B2-3). For the 3 youngest age groups, females had greater internal rotation than males; however, there was no such difference found in the other 4 age groups (Table B2-4).

### Flexion

There was a statistically significant but trivial interaction effect between age and arm (*P* < .001, η_p_^2^ = 0.007). In both the right and left arms, there were numerous differences in pairwise comparisons between age groups (Tables B3-1 and B3-2), with the younger group producing greater flexion than the older group. For the 2 youngest age groups, the left arm had greater flexion than the right arm (Table B3-3). For the next 3 age groups, the right arm had greater flexion than the left arm. For the 2 oldest age groups, there was no difference found between the arms. Differences between males and females were not statistically significant (*P* = .13).

### Extension

There was a statistically significant but trivial interaction effect between age and sex (*P* = .02, η_p_^2^ = 0.002). Among the 21 age group pairwise comparisons within males, only 3 revealed statistically significant differences (Table B4-1). Among females, no age group effect was found (Table B4-2). Although there was no statistically significant difference between sexes for the 2 oldest age groups, females had greater extension than males for the 5 younger age groups (Table B4-3).

There was also a statistically significant but trivial interaction effect between age and arm (*P* < .001, η_p_^2^ = 0.004). For the right arm, no age group effect was found (Table B4-4). Among the 21 age group pairwise comparisons for the left arm, only 3 revealed statistically significant differences (Table B4-5). Finally, although there was no significant difference between arms for the oldest age group, the right arm had greater extension for the other 6 age groups (Table B4-6).

### Abduction

Overall, there was a trend in which abduction was greater for females than males and greater for the left arm than right arm. Abduction was greatest in the 2 youngest groups. The age × sex × arm interaction effect was statistically significant but trivial (*P* = .01, η_p_^2^ = 0.003), with numerous statistical differences in pairwise comparisons (Tables B5-1 through B5-6).

### Horizontal abduction

Overall, there was a trend in which horizontal abduction was greater for females than males. There was a statistically significant but trivial interaction effect between sex and age (*P* < .001, η_p_^2^ = 0.005). Among males, horizontal abduction did not change with age (Table B6-1). In contrast, among females, horizontal abduction was greatest in the 2 youngest age groups (Tables B6-2 and B6-3).

There was also a statistically significant but trivial interaction effect between age and arm (*P* < .001, η_p_^2^ = 0.006). For both the right and left arms, horizontal abduction was greatest in the 2 youngest age groups (Tables B6-4 and B6-5). Finally, there was large inconsistency among age groups regarding which arm had greater maximum horizontal abduction angle (Table B6-6).

## Discussion

Our hypothesis that motion decreases with age was partially supported. External rotation and flexion decreased significantly with age. Abduction decreased in males from the teens into the 30s, whereas horizontal abduction decreased in females from the teens into the 30s. These results are consistent with previous studies showing decrease in shoulder active range of motion with age.^2^^,^^11^ Previous studies of passive range of motion reported decreased shoulder motion with age,^8^^,^^13^ whereas age-related changes for other joints (knee, hip, and elbow) were not significant.^13^

As hypothesized, there were several significant differences between males and females. Under age 40 years, external rotation of the right shoulder was greater for males than females; however, there were no significant differences in external rotation of the left shoulder. This contradicts previous studies, showing greater external rotation in females.^2^^,^^11^ Several other parameters in the present study were greater for females than males. Females consistently had greater abduction than their male counterparts. This is consistent with the findings of Barnes et al,^2^ whereas Gill et al reported greater abduction for males than females.^11^ Under 60 years of age, extension and horizontal abduction were greater for females than males. Under 30 years of age, internal rotation was greater for females than males.

**Figure 2:**
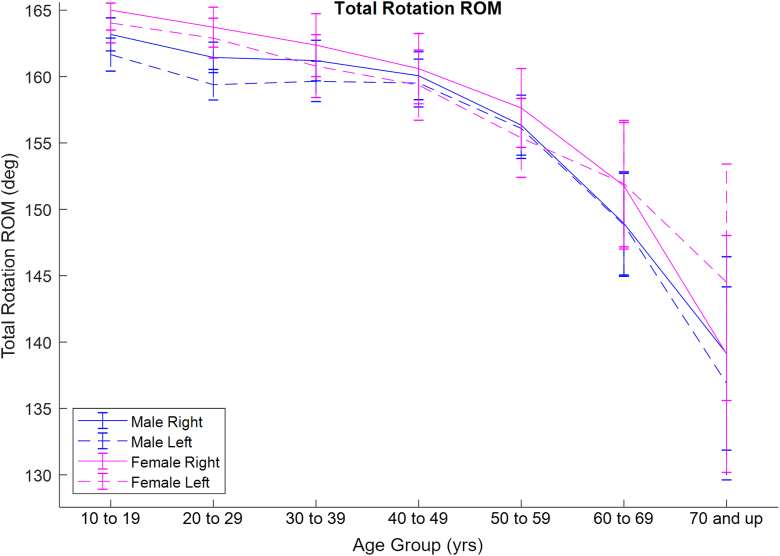
Total range of motion (ER+IR) vs. age. Blue lines correspond to males, whereas pink lines correspond to females. Solid lines correspond to right arms, whereas dashed lines correspond to left arms. Error bars denote 95% confidence intervals. *ROM*, range of motion; *ER*, external rotation; *IR*, internal rotation.

Our third hypothesis was also supported by the data, as there were significant differences between left and right shoulder range of motion. Although arm dominance was not recorded with data collection, it is reasonable to assume that the vast majority of subjects were righthanded, as approximately 90% of the general population is righthanded. In the study by Gill et al, 11% of participants were left-handed, and shoulder range of motion differences between right and left arms mirrored differences between dominant and nondominant arms.^11^ Thus, differences between right and left shoulder range of motion in the present study likely reflect differences between dominant and nondominant shoulders, respectively. External rotation was greater in the right arm, whereas internal rotation was greater in the left arm. Similar discrepancies are well documented between dominant and nondominant shoulder passive ranges of motion, especially with the throwing and nonthrowing arms of athletes.^19^^,^^23^^,^^24^ The increase in external rotation and decrease in internal rotation in the dominant arm are associated with glenoid and humeral retrotorsion, as first shown by Crockett et al.^6^ Wilk et al introduced the concept of total range of motion, defined as the summation of external rotation and internal rotation. Wilk et al have shown that although the dominant arm has greater external rotation and less internal rotation than the nondominant arm, the total passive range of motion of the 2 shoulders is about the same.^19^^,^^23^^,^^24^ Combining external rotation and internal rotation data from the present study, a post-hoc analysis of total rotation active range of motion was produced (Fig. 2). There was a significant interaction among arm, sex, and age (*P* = .03). While total active range of motion tended to be slightly greater for the right arm, this difference reached statistical significance for fewer than half of the age/sex subgroups (Table B7-6).

Differences in extension and abduction were also shown. Extension was greater in the right shoulder. For male subjects, abduction was greater in the left shoulder. Gill et al reported greater abduction in males than females,^11^ whereas Barnes et al found greater abduction in females than males.^2^

As with all studies, this investigation had limitations. This study used data from a large, heterogenous sample. Although this enabled us to establish normative data for active range of motion, future research may focus on specific groups based on recreational activities, work, and lifestyle, such as athletes, workers, or injured people. Another limitation was the omission of arm dominance. The present study found significant differences between ranges of motion of the left shoulder and right shoulder because left and right correlated to nondominant and dominant, respectively, for most people. However, knowing the arm dominance of individual would have strengthened the statistical differences even further. Statistically, the large number of comparisons analyzed created a high chance of Type I error; thus, our interpretation focused on clusters of multiple differences observed. Furthermore, this study assessed the active range of motion without controlling for motion compensations or abnormal movements.

## Conclusion

In conclusion, active range of motion of the shoulder decreases with age. The profiles of these decreases differ across specific shoulder motions. Differences were also demonstrated between sex (male/female) and arm side (right/left), although their effects were less pronounced than that of age. It is also important to note that there are numerous interactions in range of motion measurements between age, sex, and arm. This study should enhance the clinician’s appreciation and recognition of shoulder active range of motion during their clinical examinations and help establish personalized goals.

## Disclaimers

Funding: No funding was disclosed by the authors.

Conflicts of interest: Glenn S. Fleisig is a consultant for Dari Motion. The other authors, their immediate families, and any research foundation with which they are affiliated have not received any financial payments or other benefits from any commercial entity related to the subject of this article.
