# Supplementary material for: Active range of motion of the shoulder: a cross-sectional study of 6635 subjects
Source: JSES Int. 2022 Sep 30;7(1):132–7. doi: 10.1016/j.jseint.2022.09.008 (PMC9937824; doi:10.1016/j.jseint.2022.09.008)
Supplement: Appendix A [file mmc1.docx]

Appendix A: Mean value and confidence interval for each group. All values reported in degrees.

|  | Female | | Male | |
| --- | --- | --- | --- | --- |
|  | Left | Right | Left | Right |
| External Rotation | | | | |
| 10-19 years | 88.8 (87.8-89.8) | 92.2 (91.2-93.2) | 89.6 (88.8-90.4) | 94.8 (94.0-95.7) |
| 20-29 years | 87.9 (86.9-88.9) | 91.0 (90.0-92.0) | 89.8 (89.0-90.5) | 94.9 (94.1-95.6) |
| 30-39 years | 85.7 (84.2-87.3) | 88.4 (86.8-90.1) | 87.0 (86.0-88.0) | 91.7 (90.7-92.7) |
| 40-49 years | 84.2 (82.5-85.9) | 87.4 (85.6-89.2) | 84.8 (83.6-85.9) | 88.8 (87.6-90.1) |
| 50-59 years | 80.7 (78.8-82.7) | 84.3 (82.2-86.3) | 80.0 (78.5-81.5) | 84.4 (82.9-86.0) |
| 60-69 years | 77.7 (74.6-80.8) | 80.5 (77.3-83.8) | 78.0 (75.4-80.5) | 81.9 (79.3-84.6) |
| 70 years and up | 73.9 (68.1-79.8) | 74.7 (68.6-80.8) | 70.0 (65.3-74.8) | 75.0 (70.1-80.0) |
| Internal Rotation | | | | |
| 10-19 years | 75.3 (74.1-76.4) | 72.8 (71.7-73.9) | 72.1 (71.2-73.0) | 68.4 (67.5-69.3) |
| 20-29 years | 75.0 (73.9-76.1) | 72.7 (71.6-73.8) | 69.6 (68.8-70.5) | 66.6 (65.7-67.4) |
| 30-39 years | 75.1 (73.3-76.8) | 73.9 (72.2-75.7) | 72.6 (71.5-73.8) | 69.5 (68.4-70.6) |
| 40-49 years | 75.2 (73.2-77.1) | 73.2 (71.2-75.1) | 74.8 (73.4-76.1) | 71.2 (69.9-72.5) |
| 50-59 years | 74.6 (72.4-76.9) | 73.4 (71.2-75.5) | 76.1 (74.4-77.8) | 71.9 (70.3-73.6) |
| 60-69 years | 74.2 (70.7-77.8) | 71.3 (67.8-74.7) | 70.9 (68.0-73.8) | 67.0 (64.2-69.9) |
| 70 years and up | 70.6 (64.0-77.2) | 64.4 (57.9-70.9) | 66.9 (61.5-72.3) | 64.1 (58.8-69.4) |
| Flexion | | | | |
| 10-19 years | 183.0 (181.9-184.0) | 182.4 (181.3-183.4) | 183.7 (182.8-184.5) | 183.0 (182.1-183.8) |
| 20-29 years | 181.6 (180.5-182.6) | 180.8 (179.7-181.8) | 183.0 (182.2-183.7) | 182.3 (181.5-183.1) |
| 30-39 years | 179.3 (177.6-180.9) | 179.6 (178.0-181.3) | 179.9 (178.9-181.0) | 181.2 (180.2-182.3) |
| 40-49 years | 176.1 (174.3-177.9) | 177.1 (175.2-178.9) | 178.2 (176.9-179.4) | 179.2 (178.0-180.5) |
| 50-59 years | 172.5 (170.5-174.5) | 173.9 (171.8-176.0) | 174.2 (172.7-175.8) | 175.0 (173.5-176.6) |
| 60-69 years | 166.7 (163.4-169.9) | 167.5 (164.2-170.8) | 166.3 (163.7-169.0) | 167.2 (164.5-169.9) |
| 70 years and up | 159.9 (153.8-166.0) | 163.7 (157.5-169.9) | 163.7 (158.8-168.7) | 161.5 (156.5-166.6) |
| Extension | | | | |
| 10-19 years | 54.9 (53.9-56.0) | 55.8 (54.8-56.8) | 50.7 (49.8-51.5) | 51.2 (50.3-52.1) |
| 20-29 years | 55.3 (54.2-56.3) | 56.3 (55.3-57.4) | 49.2 (48.4-49.9) | 50.2 (49.4-51.0) |
| 30-39 years | 53.2 (51.6-54.9) | 54.9 (53.3-56.5) | 46.1 (45.1-47.2) | 48.6 (47.5-49.7) |
| 40-49 years | 54.0 (52.1-55.8) | 56.0 (54.1-57.8) | 47.0 (45.7-48.2) | 49.1 (47.8-50.3) |
| 50-59 years | 54.4 (52.4-56.4) | 55.5 (53.4-57.5) | 48.5 (46.9-50.1) | 50.1 (48.5-51.7) |
| 60-69 years | 51.5 (48.2-54.8) | 53.1 (49.8-56.4) | 51.1 (48.5-53.8) | 52.0 (49.3-54.7) |
| 70 years and up | 49.0 (42.9-55.2) | 49.4 (43.2-55.6) | 49.7 (44.7-54.7) | 51.1 (46.0-56.1) |
| Abduction | | | | |
| 10-19 years | 183.4 (182.6-184.1) | 182.3 (181.5-183.1) | 182.8 (182.1-183.4) | 181.5 (180.9-182.2) |
| 20-29 years | 182.0 (181.2-182.7) | 181.4 (180.7-182.2) | 179.1 (178.5-179.7) | 177.8 (177.2-178.4) |
| 30-39 years | 182.0 (180.8-183.2) | 180.3 (179.1-181.6) | 175.6 (174.8-176.4) | 173.5 (172.7-174.3) |
| 40-49 years | 179.8 (178.4-181.1) | 179.9 (178.6-181.3) | 175.8 (174.8-176.7) | 173.3 (172.4-174.3) |
| 50-59 years | 180.6 (179.1-182.1) | 179.6 (178.1-181.2) | 177.0 (175.8-178.1) | 173.7 (172.6-174.9) |
| 60-69 years | 178.9 (176.5-181.4) | 179.8 (177.3-182.3) | 177.1 (175.1-179.1) | 173.4 (171.4-175.4) |
| 70 years and up | 180.3 (175.7-184.8) | 177.8 (173.2-182.4) | 173.5 (169.8-177.2) | 175.2 (171.4-179.0) |
| Horizontal Abduction | | | | |
| 10-19 years | 49.4 (48.2-50.6) | 47.5 (46.4-48.7) | 40.5 (39.5-41.6) | 38.6 (37.6-39.5) |
| 20-29 years | 48.1 (46.9-49.3) | 47.0 (45.9-48.1) | 39.9 (39.0-40.9) | 39.3 (38.4-40.2) |
| 30-39 years | 42.7 (40.8-44.6) | 42.2 (40.5-44.0) | 37.9 (36.7-39.1) | 37.9 (36.7-39.0) |
| 40-49 years | 41.5 (39.4-43.6) | 42.5 (40.5-44.5) | 37.8 (36.3-39.2) | 38.8 (37.4-40.2) |
| 50-59 years | 41.8 (39.4-44.2) | 40.0 (37.7-42.2) | 37.3 (35.5-39.2) | 36.0 (34.3-37.7) |
| 60-69 years | 41.5 (37.7-45.4) | 39.4 (35.8-43.0) | 38.0 (34.9-41.1) | 38.1 (35.1-41.0) |
| 70 years and up | 42.2 (35.0-49.4) | 35.4 (28.7-42.2) | 43.8 (37.9-49.6) | 38.9 (33.4-44.4) |
