# Supplementary material for: Active range of motion of the shoulder: a cross-sectional study of 6635 subjects
Source: JSES Int. 2022 Sep 30;7(1):132–7. doi: 10.1016/j.jseint.2022.09.008 (PMC9937824; doi:10.1016/j.jseint.2022.09.008)
Supplement: Appendix B [file mmc2.docx]

Appendix B. Statistical pairwise comparison tables.

**External rotation**

Table B1-1. Results of pairwise comparisons between age groups for maximum external rotation (for all sexes and arms). ‘Y’ denotes a comparison where the younger group had statistically significantly greater value than the older group (p<0.05). ‘-’ denotes no difference found. Cells for duplicate comparisons have been intentionally left blank.

| **Age Group** | **10-to-19** | **20-to-29** | **30-to-39** | **40-to-49** | **50-to-59** | **60-to-69** |  |
| --- | --- | --- | --- | --- | --- | --- | --- |
| **20-to-29** | - |  |  |  |  |  | **20-to-29** |
| **30-to-39** | Y | Y |  |  |  |  | **30-to-39** |
| **40-to-49** | Y | Y | - |  |  |  | **40-to-49** |
| **50-to-59** | Y | Y | Y | Y |  |  | **50-to-59** |
| **60-to-69** | Y | Y | Y | Y | - |  | **60-to-69** |
| **70-and-up** | Y | Y | Y | Y | Y | - | **70-and-up** |
|  | **10-to-19** | **20-to-29** | **30-to-39** | **40-to-49** | **50-to-59** | **60-to-69** |  |

Table B1-2. Results of pairwise comparisons between sex for maximum external rotation (performed for each arm separately due to interaction effect). ‘M’ denotes a comparison where males had statistically significantly greater value than females (p<0.05). ‘-’ denotes no difference found.

| **Side** | **Right** | **Left** |
| --- | --- | --- |
| **Sex with greater value** | M | - |

Table B1-3. Results of pairwise comparisons between arm side for maximum external rotation (performed for each sex separately due to interaction effect). ‘R’ denotes a comparison where the right arm had statistically significantly greater value than the left arm (p<0.05).

| **Side** | **Right** | **Left** |
| --- | --- | --- |
| **Side with greater value** | R | R |

**Internal rotation**

Table B2-1. Results of pairwise comparisons between arm side for maximum external rotation (for all ages and sexes). ‘L’ denotes a comparison where the left arm had statistically significantly greater value than the right arm (p<0.05).

|  | **All Ages and Sexes** |
| --- | --- |
| **Side with greater value** | L |

Table B2-2. Results of pairwise comparisons between age groups for maximum internal rotation (for males). ‘Y’ denotes a comparison where the younger group had statistically significantly greater value than the older group (p<0.05), while ‘O’ denotes the opposite. ‘-’ denotes no difference found. Cells for duplicate comparisons have been intentionally left blank.

| **Age Group** | **10-to-19** | **20-to-29** | **30-to-39** | **40-to-49** | **50-to-59** | **60-to-69** |  |
| --- | --- | --- | --- | --- | --- | --- | --- |
| **20-to-29** | Y |  |  |  |  |  | **20-to-29** |
| **30-to-39** | - | O |  |  |  |  | **30-to-39** |
| **40-to-49** | O | O | - |  |  |  | **40-to-49** |
| **50-to-59** | O | O | - | - |  |  | **50-to-59** |
| **60-to-69** | - | - | - | - | Y |  | **60-to-69** |
| **70-and-up** | - | - | - | - | Y | - | **70-and-up** |
|  | **10-to-19** | **20-to-29** | **30-to-39** | **40-to-49** | **50-to-59** | **60-to-69** |  |

Table B2-3. Results of pairwise comparisons between age groups for maximum internal rotation (for females). ‘-’ denotes no difference found (p>0.05). Cells for duplicate comparisons have been intentionally left blank.

| **Age Group** | **10-to-19** | **20-to-29** | **30-to-39** | **40-to-49** | **50-to-59** | **60-to-69** |  |
| --- | --- | --- | --- | --- | --- | --- | --- |
| **20-to-29** | - |  |  |  |  |  | **20-to-29** |
| **30-to-39** | - | - |  |  |  |  | **30-to-39** |
| **40-to-49** | - | - | - |  |  |  | **40-to-49** |
| **50-to-59** | - | - | - | - |  |  | **50-to-59** |
| **60-to-69** | - | - | - | - | - |  | **60-to-69** |
| **70-and-up** | - | - | - | - | - | - | **70-and-up** |
|  | **10-to-19** | **20-to-29** | **30-to-39** | **40-to-49** | **50-to-59** | **60-to-69** |  |

Table B2-4. Results of pairwise comparisons between sex for maximum internal rotation (performed for each age group separately due to interaction effect). ‘F’ denotes a comparison where females had statistically significantly greater value than males (p<0.05). ‘-’ denotes no difference found.

| **Age Group** | **10-to-19** | **20-to-29** | **30-to-39** | **40-to-49** | **50-to-59** | **60-to-69** | **70-and-up** |
| --- | --- | --- | --- | --- | --- | --- | --- |
| **Sex with greater value** | F | F | F | - | - | - | - |

**Flexion**

Table B3-1. Results of pairwise comparisons between age groups for maximum flexion (for right arms). ‘Y’ denotes a comparison where the younger group had statistically significantly greater value than the older group (p<0.05). ‘-’ denotes no difference found. Cells for duplicate comparisons have been intentionally left blank.

| **Age Group** | **10-to-19** | **20-to-29** | **30-to-39** | **40-to-49** | **50-to-59** | **60-to-69** |  |
| --- | --- | --- | --- | --- | --- | --- | --- |
| **20-to-29** | - |  |  |  |  |  | **20-to-29** |
| **30-to-39** | Y | - |  |  |  |  | **30-to-39** |
| **40-to-49** | Y | Y | - |  |  |  | **40-to-49** |
| **50-to-59** | Y | Y | Y | Y |  |  | **50-to-59** |
| **60-to-69** | Y | Y | Y | Y | Y |  | **60-to-69** |
| **70-and-up** | Y | Y | Y | Y | Y | - | **70-and-up** |
|  | **10-to-19** | **20-to-29** | **30-to-39** | **40-to-49** | **50-to-59** | **60-to-69** |  |

Table B3-2. Results of pairwise comparisons between age groups for maximum flexion (for left arms). ‘Y’ denotes a comparison where the younger group had statistically significantly greater value than the older group (p<0.05). ‘-’ denotes no difference found. Cells for duplicate comparisons have been intentionally left blank.

| **Age Group** | **10-to-19** | **20-to-29** | **30-to-39** | **40-to-49** | **50-to-59** | **60-to-69** |  |
| --- | --- | --- | --- | --- | --- | --- | --- |
| **20-to-29** | - |  |  |  |  |  | **20-to-29** |
| **30-to-39** | Y | Y |  |  |  |  | **30-to-39** |
| **40-to-49** | Y | Y | Y |  |  |  | **40-to-49** |
| **50-to-59** | Y | Y | Y | Y |  |  | **50-to-59** |
| **60-to-69** | Y | Y | Y | Y | Y |  | **60-to-69** |
| **70-and-up** | Y | Y | Y | Y | Y | - | **70-and-up** |
|  | **10-to-19** | **20-to-29** | **30-to-39** | **40-to-49** | **50-to-59** | **60-to-69** |  |

Table B3-3. Results of pairwise comparisons between arm side for maximum flexion (performed for each age group separately due to interaction effect). ‘R’ denotes a comparison where the right arm had statistically significantly greater value than the left arm (p<0.05), while ‘L’ denotes the opposite. ‘-’ denotes no difference found.

| **Age Group** | **10-to-19** | **20-to-29** | **30-to-39** | **40-to-49** | **50-to-59** | **60-to-69** | **70-and-up** |
| --- | --- | --- | --- | --- | --- | --- | --- |
| **Side with greater value** | L | L | R | R | R | - | - |

**Extension**

Table B4-1. Results of pairwise comparisons between age groups for maximum extension (for males). ‘Y’ denotes a comparison where the younger group had statistically significantly greater value than the older group (p<0.05). ‘-’ denotes no difference found. Cells for duplicate comparisons have been intentionally left blank.

| **Age Group** | **10-to-19** | **20-to-29** | **30-to-39** | **40-to-49** | **50-to-59** | **60-to-69** |  |
| --- | --- | --- | --- | --- | --- | --- | --- |
| **20-to-29** | - |  |  |  |  |  | **20-to-29** |
| **30-to-39** | Y | Y |  |  |  |  | **30-to-39** |
| **40-to-49** | Y | - | - |  |  |  | **40-to-49** |
| **50-to-59** | - | - | - | - |  |  | **50-to-59** |
| **60-to-69** | - | - | - | - | - |  | **60-to-69** |
| **70-and-up** | - | - | - | - | - | - | **70-and-up** |
|  | **10-to-19** | **20-to-29** | **30-to-39** | **40-to-49** | **50-to-59** | **60-to-69** |  |

Table B4-2. Results of pairwise comparisons between age groups for maximum internal rotation (for females). ‘-’ denotes no difference found (p>0.05). Cells for duplicate comparisons have been intentionally left blank.

| **Age Group** | **10-to-19** | **20-to-29** | **30-to-39** | **40-to-49** | **50-to-59** | **60-to-69** |  |
| --- | --- | --- | --- | --- | --- | --- | --- |
| **20-to-29** | - |  |  |  |  |  | **20-to-29** |
| **30-to-39** | - | - |  |  |  |  | **30-to-39** |
| **40-to-49** | - | - | - |  |  |  | **40-to-49** |
| **50-to-59** | - | - | - | - |  |  | **50-to-59** |
| **60-to-69** | - | - | - | - | - |  | **60-to-69** |
| **70-and-up** | - | - | - | - | - | - | **70-and-up** |
|  | **10-to-19** | **20-to-29** | **30-to-39** | **40-to-49** | **50-to-59** | **60-to-69** |  |

Table B4-3. Results of pairwise comparisons between sex for maximum extension (performed for each age group separately due to interaction effect). ‘F’ denotes a comparison where females had statistically significantly greater value than males (p<0.05). ‘-’ denotes no difference found.

| **Age Group** | **10-to-19** | **20-to-29** | **30-to-39** | **40-to-49** | **50-to-59** | **60-to-69** | **70-and-up** |
| --- | --- | --- | --- | --- | --- | --- | --- |
| **Sex with greater value** | F | F | F | F | F | - | - |

Table B4-4. Results of pairwise comparisons between age groups for maximum extension (for right arms). ‘-’ denotes no difference found (p>0.05). Cells for duplicate comparisons have been intentionally left blank.

| **Age Group** | **10-to-19** | **20-to-29** | **30-to-39** | **40-to-49** | **50-to-59** | **60-to-69** |  |
| --- | --- | --- | --- | --- | --- | --- | --- |
| **20-to-29** | - |  |  |  |  |  | **20-to-29** |
| **30-to-39** | - | - |  |  |  |  | **30-to-39** |
| **40-to-49** | - | - | - |  |  |  | **40-to-49** |
| **50-to-59** | - | - | - | - |  |  | **50-to-59** |
| **60-to-69** | - | - | - | - | - |  | **60-to-69** |
| **70-and-up** | - | - | - | - | - | - | **70-and-up** |
|  | **10-to-19** | **20-to-29** | **30-to-39** | **40-to-49** | **50-to-59** | **60-to-69** |  |

Table B4-5. Results of pairwise comparisons between age groups for maximum extension (for left arms). ‘Y’ denotes a comparison where the younger group had statistically significantly greater value than the older group (p<0.05). ‘-’ denotes no difference found. Cells for duplicate comparisons have been intentionally left blank.

| **Age Group** | **10-to-19** | **20-to-29** | **30-to-39** | **40-to-49** | **50-to-59** | **60-to-69** |  |
| --- | --- | --- | --- | --- | --- | --- | --- |
| **20-to-29** | - |  |  |  |  |  | **20-to-29** |
| **30-to-39** | Y | Y |  |  |  |  | **30-to-39** |
| **40-to-49** | Y | - | - |  |  |  | **40-to-49** |
| **50-to-59** | - | - | - | - |  |  | **50-to-59** |
| **60-to-69** | - | - | - | - | - |  | **60-to-69** |
| **70-and-up** | - | - | - | - | - | - | **70-and-up** |
|  | **10-to-19** | **20-to-29** | **30-to-39** | **40-to-49** | **50-to-59** | **60-to-69** |  |

Table B4-6. Results of pairwise comparisons between arm side for maximum extension (performed for each age group separately due to interaction effect). ‘R’ denotes a comparison where the right arm had statistically significantly greater value than the left arm (p<0.05). ‘-’ denotes no difference found.

| **Age Group** | **10-to-19** | **20-to-29** | **30-to-39** | **40-to-49** | **50-to-59** | **60-to-69** | **70-and-up** |
| --- | --- | --- | --- | --- | --- | --- | --- |
| **Side with greater value** | R | R | R | R | R | R | - |

**Abduction**

Table B5-1. Results of pairwise comparisons between age groups for maximum abduction (for the right arms of males). ‘Y’ denotes a comparison where the younger group had statistically significantly greater value than the older group (p<0.05). ‘-’ denotes no difference found. Cells for duplicate comparisons have been intentionally left blank.

| **Age Group** | **10-to-19** | **20-to-29** | **30-to-39** | **40-to-49** | **50-to-59** | **60-to-69** |  |
| --- | --- | --- | --- | --- | --- | --- | --- |
| **20-to-29** | Y |  |  |  |  |  | **20-to-29** |
| **30-to-39** | Y | Y |  |  |  |  | **30-to-39** |
| **40-to-49** | Y | Y | - |  |  |  | **40-to-49** |
| **50-to-59** | Y | Y | - | - |  |  | **50-to-59** |
| **60-to-69** | Y | Y | - | - | - |  | **60-to-69** |
| **70-and-up** | Y | - | - | - | - | - | **70-and-up** |
|  | **10-to-19** | **20-to-29** | **30-to-39** | **40-to-49** | **50-to-59** | **60-to-69** |  |

Table B5-2. Results of pairwise comparisons between age groups for maximum abduction (for the left arms of males). ‘Y’ denotes a comparison where the younger group had statistically significantly greater value than the older group (p<0.05). ‘-’ denotes no difference found. Cells for duplicate comparisons have been intentionally left blank.

| **Age Group** | **10-to-19** | **20-to-29** | **30-to-39** | **40-to-49** | **50-to-59** | **60-to-69** |  |
| --- | --- | --- | --- | --- | --- | --- | --- |
| **20-to-29** | Y |  |  |  |  |  | **20-to-29** |
| **30-to-39** | Y | Y |  |  |  |  | **30-to-39** |
| **40-to-49** | Y | Y | - |  |  |  | **40-to-49** |
| **50-to-59** | Y | Y | - | - |  |  | **50-to-59** |
| **60-to-69** | Y | - | - | - | - |  | **60-to-69** |
| **70-and-up** | Y | - | - | - | - | - | **70-and-up** |
|  | **10-to-19** | **20-to-29** | **30-to-39** | **40-to-49** | **50-to-59** | **60-to-69** |  |

Table B5-3. Results of pairwise comparisons between age groups for maximum abduction (for the right arms of females). ‘Y’ denotes a comparison where the younger group had statistically significantly greater value than the older group (p<0.05). ‘-’ denotes no difference found. Cells for duplicate comparisons have been intentionally left blank.

| **Age Group** | **10-to-19** | **20-to-29** | **30-to-39** | **40-to-49** | **50-to-59** | **60-to-69** |  |
| --- | --- | --- | --- | --- | --- | --- | --- |
| **20-to-29** | - |  |  |  |  |  | **20-to-29** |
| **30-to-39** | - | - |  |  |  |  | **30-to-39** |
| **40-to-49** | - | - | - |  |  |  | **40-to-49** |
| **50-to-59** | Y | - | - | - |  |  | **50-to-59** |
| **60-to-69** | - | - | - | - | - |  | **60-to-69** |
| **70-and-up** | - | - | - | - | - | - | **70-and-up** |
|  | **10-to-19** | **20-to-29** | **30-to-39** | **40-to-49** | **50-to-59** | **60-to-69** |  |

Table B5-4. Results of pairwise comparisons between age groups for maximum abduction (for the left arms of females). ‘Y’ denotes a comparison where the younger group had statistically significantly greater value than the older group (p<0.05). ‘-’ denotes no difference found. Cells for duplicate comparisons have been intentionally left blank.

| **Age Group** | **10-to-19** | **20-to-29** | **30-to-39** | **40-to-49** | **50-to-59** | **60-to-69** |  |
| --- | --- | --- | --- | --- | --- | --- | --- |
| **20-to-29** | - |  |  |  |  |  | **20-to-29** |
| **30-to-39** | - | - |  |  |  |  | **30-to-39** |
| **40-to-49** | Y | - | - |  |  |  | **40-to-49** |
| **50-to-59** | Y | - | - | - |  |  | **50-to-59** |
| **60-to-69** | Y | - | - | - | - |  | **60-to-69** |
| **70-and-up** | - | - | - | - | - | - | **70-and-up** |
|  | **10-to-19** | **20-to-29** | **30-to-39** | **40-to-49** | **50-to-59** | **60-to-69** |  |

Table B5-5. Results of pairwise comparisons between sex for maximum abduction (performed for each age*arm subgroup separately due to interaction effect). ‘F’ denotes a comparison where females had statistically significantly greater value than males (p<0.05). ‘-’ denotes no difference found.

| **Age Group** | **10-to-19** | **20-to-29** | **30-to-39** | **40-to-49** | **50-to-59** | **60-to-69** | **70-and-up** |
| --- | --- | --- | --- | --- | --- | --- | --- |
| **Right** | - | F | F | F | F | F | - |
| **Left** | - | F | F | F | F | - | F |

Table B5-6. Results of pairwise comparisons between arm side for maximum abduction (performed for each age*sex subgroup separately due to interaction effect). ‘L’ denotes a comparison where the left arm had statistically significantly greater value than the right arm (p<0.05). ‘-’ denotes no difference found.

| **Age Group** | **10-to-19** | **20-to-29** | **30-to-39** | **40-to-49** | **50-to-59** | **60-to-69** | **70-and-up** |
| --- | --- | --- | --- | --- | --- | --- | --- |
| **Male** | L | L | L | L | L | L | - |
| **Female** | L | - | L | - | - | - | - |

**Horizontal Abduction**

Table B6-1. Results of pairwise comparisons between age groups for maximum extension (for males). ‘Y’ denotes a comparison where the younger group had statistically significantly greater value than the older group (p<0.05). ‘-’ denotes no difference found. Cells for duplicate comparisons have been intentionally left blank.

| **Age Group** | **10-to-19** | **20-to-29** | **30-to-39** | **40-to-49** | **50-to-59** | **60-to-69** |  |
| --- | --- | --- | --- | --- | --- | --- | --- |
| **20-to-29** | - |  |  |  |  |  | **20-to-29** |
| **30-to-39** | - | - |  |  |  |  | **30-to-39** |
| **40-to-49** | - | - | - |  |  |  | **40-to-49** |
| **50-to-59** | - | Y | - | - |  |  | **50-to-59** |
| **60-to-69** | - | - | - | - | - |  | **60-to-69** |
| **70-and-up** | - | - | - | - | - | - | **70-and-up** |
|  | **10-to-19** | **20-to-29** | **30-to-39** | **40-to-49** | **50-to-59** | **60-to-69** |  |

Table B6-2. Results of pairwise comparisons between age groups for maximum internal rotation (for females). ‘Y’ denotes a comparison where the younger group had statistically significantly greater value than the older group (p<0.05). ‘-’ denotes no difference found. Cells for duplicate comparisons have been intentionally left blank.

| **Age Group** | **10-to-19** | **20-to-29** | **30-to-39** | **40-to-49** | **50-to-59** | **60-to-69** |  |
| --- | --- | --- | --- | --- | --- | --- | --- |
| **20-to-29** | - |  |  |  |  |  | **20-to-29** |
| **30-to-39** | Y | Y |  |  |  |  | **30-to-39** |
| **40-to-49** | Y | Y | - |  |  |  | **40-to-49** |
| **50-to-59** | Y | Y | - | - |  |  | **50-to-59** |
| **60-to-69** | Y | Y | - | - | - |  | **60-to-69** |
| **70-and-up** | - | - | - | - | - | - | **70-and-up** |
|  | **10-to-19** | **20-to-29** | **30-to-39** | **40-to-49** | **50-to-59** | **60-to-69** |  |

Table B6-3. Results of pairwise comparisons between sex for maximum horizontal abduction (performed for each age group separately due to interaction effect). ‘F’ denotes a comparison where females had statistically significantly greater value than males (p<0.05). ‘-’ denotes no difference found.

| **Age Group** | **10-to-19** | **20-to-29** | **30-to-39** | **40-to-49** | **50-to-59** | **60-to-69** | **70-and-up** |
| --- | --- | --- | --- | --- | --- | --- | --- |
| **Sex with greater value** | F | F | F | F | F | - | - |

Table B6-4. Results of pairwise comparisons between age groups for maximum horizontal abduction (for right arms). ‘Y’ denotes a comparison where the younger group had statistically significantly greater value than the older group (p<0.05). ‘-’ denotes no difference found. Cells for duplicate comparisons have been intentionally left blank.

| **Age Group** | **10-to-19** | **20-to-29** | **30-to-39** | **40-to-49** | **50-to-59** | **60-to-69** |  |
| --- | --- | --- | --- | --- | --- | --- | --- |
| **20-to-29** | - |  |  |  |  |  | **20-to-29** |
| **30-to-39** | Y | Y |  |  |  |  | **30-to-39** |
| **40-to-49** | Y | Y | - |  |  |  | **40-to-49** |
| **50-to-59** | Y | Y | - | - |  |  | **50-to-59** |
| **60-to-69** | Y | Y | - | - | - |  | **60-to-69** |
| **70-and-up** | - | - | - | - | - | - | **70-and-up** |
|  | **10-to-19** | **20-to-29** | **30-to-39** | **40-to-49** | **50-to-59** | **60-to-69** |  |

Table B6-5. Results of pairwise comparisons between age groups for maximum horizontal abduction (for left arms). ‘Y’ denotes a comparison where the younger group had statistically significantly greater value than the older group (p<0.05). ‘-’ denotes no difference found. Cells for duplicate comparisons have been intentionally left blank.

| **Age Group** | **10-to-19** | **20-to-29** | **30-to-39** | **40-to-49** | **50-to-59** | **60-to-69** |  |
| --- | --- | --- | --- | --- | --- | --- | --- |
| **20-to-29** | - |  |  |  |  |  | **20-to-29** |
| **30-to-39** | Y | Y |  |  |  |  | **30-to-39** |
| **40-to-49** | Y | Y | - |  |  |  | **40-to-49** |
| **50-to-59** | Y | Y | - | - |  |  | **50-to-59** |
| **60-to-69** | Y | Y | - | - | - |  | **60-to-69** |
| **70-and-up** | - | - | - | - | - | - | **70-and-up** |
|  | **10-to-19** | **20-to-29** | **30-to-39** | **40-to-49** | **50-to-59** | **60-to-69** |  |

Table B6-6. Results of pairwise comparisons between arm side for maximum horizontal abduction (performed for each age group separately due to interaction effect). ‘R’ denotes a comparison where the right arm had statistically significantly greater value than the left arm (p<0.05), while ‘L’ denotes the opposite. ‘-’ denotes no difference found.

| **Age Group** | **10-to-19** | **20-to-29** | **30-to-39** | **40-to-49** | **50-to-59** | **60-to-69** | **70-and-up** |
| --- | --- | --- | --- | --- | --- | --- | --- |
| **Side with greater value** | L | L | - | R | L | - | L |

**Posthoc Analyses - Total Rotation ROM (External + Internal)**

Table B7-1. Results of pairwise comparisons between age groups for total rotation range of motion (for the right arms of males). ‘Y’ denotes a comparison where the younger group had statistically significantly greater value than the older group (p<0.05). ‘-’ denotes no difference found. Cells for duplicate comparisons have been intentionally left blank.

| **Age Group** | **10-to-19** | **20-to-29** | **30-to-39** | **40-to-49** | **50-to-59** | **60-to-69** |  |
| --- | --- | --- | --- | --- | --- | --- | --- |
| **20-to-29** | - |  |  |  |  |  | **20-to-29** |
| **30-to-39** | - | - |  |  |  |  | **30-to-39** |
| **40-to-49** | - | - | - |  |  |  | **40-to-49** |
| **50-to-59** | Y | Y | Y | - |  |  | **50-to-59** |
| **60-to-69** | Y | Y | Y | Y | Y |  | **60-to-69** |
| **70-and-up** | Y | Y | Y | Y | Y | - | **70-and-up** |
|  | **10-to-19** | **20-to-29** | **30-to-39** | **40-to-49** | **50-to-59** | **60-to-69** |  |

Table B7-2. Results of pairwise comparisons between age groups for total rotation range of motion (for the left arms of males). ‘Y’ denotes a comparison where the younger group had statistically significantly greater value than the older group (p<0.05). ‘-’ denotes no difference found. Cells for duplicate comparisons have been intentionally left blank.

| **Age Group** | **10-to-19** | **20-to-29** | **30-to-39** | **40-to-49** | **50-to-59** | **60-to-69** |  |
| --- | --- | --- | --- | --- | --- | --- | --- |
| **20-to-29** | - |  |  |  |  |  | **20-to-29** |
| **30-to-39** | - | - |  |  |  |  | **30-to-39** |
| **40-to-49** | - | - | - |  |  |  | **40-to-49** |
| **50-to-59** | Y | - | - | - |  |  | **50-to-59** |
| **60-to-69** | Y | Y | Y | Y | Y |  | **60-to-69** |
| **70-and-up** | Y | Y | Y | Y | Y | - | **70-and-up** |
|  | **10-to-19** | **20-to-29** | **30-to-39** | **40-to-49** | **50-to-59** | **60-to-69** |  |

Table B7-3. Results of pairwise comparisons between age groups for total rotation range of motion (for the right arms of females). ‘Y’ denotes a comparison where the younger group had statistically significantly greater value than the older group (p<0.05). ‘-’ denotes no difference found. Cells for duplicate comparisons have been intentionally left blank.

| **Age Group** | **10-to-19** | **20-to-29** | **30-to-39** | **40-to-49** | **50-to-59** | **60-to-69** |  |
| --- | --- | --- | --- | --- | --- | --- | --- |
| **20-to-29** | - |  |  |  |  |  | **20-to-29** |
| **30-to-39** | - | - |  |  |  |  | **30-to-39** |
| **40-to-49** | - | - | - |  |  |  | **40-to-49** |
| **50-to-59** | Y | Y | - | - |  |  | **50-to-59** |
| **60-to-69** | Y | Y | Y | Y | - |  | **60-to-69** |
| **70-and-up** | Y | Y | Y | Y | Y | - | **70-and-up** |
|  | **10-to-19** | **20-to-29** | **30-to-39** | **40-to-49** | **50-to-59** | **60-to-69** |  |

Table B7-4. Results of pairwise comparisons between age groups for total rotation range of motion (for the left arms of females). ‘Y’ denotes a comparison where the younger group had statistically significantly greater value than the older group (p<0.05). ‘-’ denotes no difference found. Cells for duplicate comparisons have been intentionally left blank.

| **Age Group** | **10-to-19** | **20-to-29** | **30-to-39** | **40-to-49** | **50-to-59** | **60-to-69** |  |
| --- | --- | --- | --- | --- | --- | --- | --- |
| **20-to-29** | - |  |  |  |  |  | **20-to-29** |
| **30-to-39** | - | - |  |  |  |  | **30-to-39** |
| **40-to-49** | - | - | - |  |  |  | **40-to-49** |
| **50-to-59** | Y | Y | - | - |  |  | **50-to-59** |
| **60-to-69** | Y | Y | Y | - | - |  | **60-to-69** |
| **70-and-up** | Y | Y | Y | Y | - | - | **70-and-up** |
|  | **10-to-19** | **20-to-29** | **30-to-39** | **40-to-49** | **50-to-59** | **60-to-69** |  |

Table B7-5. Results of pairwise comparisons between sex for total rotation range of motion (performed for each age*arm subgroup separately due to interaction effect). ‘F’ denotes a comparison where females had statistically significantly greater value than males (p<0.05). ‘-’ denotes no difference found.

| **Age Group** | **10-to-19** | **20-to-29** | **30-to-39** | **40-to-49** | **50-to-59** | **60-to-69** | **70-and-up** |
| --- | --- | --- | --- | --- | --- | --- | --- |
| **Right** | - | F | - | - | - | - | - |
| **Left** | F | F | - | - | - | - | - |

Table B7-6. Results of pairwise comparisons between arm side for total rotation range of motion (performed for each age*sex subgroup separately due to interaction effect). ‘R’ (‘L’) denotes a comparison where the right (left) arm had statistically significantly greater value than the right (left) arm (p<0.05). ‘-’ denotes no difference found.

| **Age Group** | **10-to-19** | **20-to-29** | **30-to-39** | **40-to-49** | **50-to-59** | **60-to-69** | **70-and-up** |
| --- | --- | --- | --- | --- | --- | --- | --- |
| **Male** | R | R | R | - | - | - | - |
| **Female** | R | - | R | - | R | - | L |
